# Factors Affecting Harp Seal (*Pagophilus groenlandicus*) Strandings in the Northwest Atlantic

**DOI:** 10.1371/journal.pone.0068779

**Published:** 2013-07-17

**Authors:** Brianne K. Soulen, Kristina Cammen, Thomas F. Schultz, David W. Johnston

**Affiliations:** 1 Duke University Marine Laboratory, Division of Marine Science and Conservation, Nicholas School of the Environment, Duke University, Beaufort, North Carolina, United States of America; 2 Marine Conservation Molecular Facility, Duke University Marine Laboratory, Division of Marine Science and Conservation, Nicholas School of the Environment, Duke University, Beaufort, North Carolina, United States of America; The Ohio State University, United States of America

## Abstract

The effects of climate change on high latitude regions are becoming increasingly evident, particularly in the rapid decline of sea ice cover in the Arctic. Many high latitude species dependent on sea ice are being forced to adapt to changing habitats. Harp seals (*Pagophilus groenlandicus*) are an indicator species for changing high-latitude ecosystems. This study analyzed multiple factors including ice cover, demographics, and genetic diversity, which could affect harp seal stranding rates along the eastern coast of the United States. Ice cover assessments were conducted for the month of February in the Gulf of St. Lawrence whelping region from 1991–2010 using remote sensing data, and harp seal stranding data were collected over the same time period. Genetic diversity, which may affect how quickly species can adapt to changing climates, was assessed using ten microsatellite markers to determine mean *d* ^2^ in a subset of stranded and by-caught (presumably healthy) seals sampled along the northeast U.S. coast. Our study found a strong negative correlation (*R* ^2^ = 0.49) between ice cover in the Gulf of St. Lawrence and yearling harp seal strandings, but found no relationship between sea ice conditions and adult strandings. Our analysis revealed that male seals stranded more frequently than females during the study period and that this relationship was strongest during light ice years. In contrast, we found no significant difference in mean *d* ^2^ between stranded and by-caught harp seals. The results demonstrate that sea ice cover and demographic factors have a greater influence on harp seal stranding rates than genetic diversity, with only a little of the variance in mean *d* ^2^ among stranded seals explained by ice cover. Any changes in these factors could have major implications for harp seals, and these findings should be considered in the development of future management plans for the Arctic that incorporate climate variability.

## Introduction

We are currently witnessing significant and rapid environmental changes in high latitude ecosystems. Climate variability is affecting sea ice dynamics in both Arctic [1] and sub-Arctic regions [2] of North America. Some predictions indicate that circumpolar sea ice cover in the Arctic may decline as much as 20% by 2050 [3] with ice-free summers starting as early as 2037 [1]. More recent studies indicate that observed changes in sea ice are outpacing model predictions [4].

Rapid changes in temperature and ice conditions can pose significant challenges for marine mammals that use sea ice as a platform for breeding and social activity. These species have evolved complex life history strategies that exploit the resources available in these systems [2], [5]. Pagophilic, or ice-loving, seals like harp seals (*Pagophilus groenlandicus*) use seasonal sea ice to give birth to and nurse their pups. Harp seals breed in two main regions, the Northwest Atlantic off the eastern coast of Canada and the Northeast Atlantic on the West Ice and in the White Sea [6]. The Northwest Atlantic population of approximately 6.9 million seals [7] breeds in two main whelping patches, the Front off Newfoundland and Labrador and in the Gulf of St. Lawrence [6], [8], [9]. Harp seal pups are born on ice in this region from late February until March and normally weaned within two weeks of birth [6], [10]. The pups generally stay on the ice until they molt their white coat, during which time they are vulnerable to predation, melting ice and human hunters [6]. After pupping and weaning, harp seals complete an annual migration northward from winter whelping grounds to summer feeding grounds that tends to follow pack ice retreat [8].

Previous studies indicate that climate variability can have significant effects on the breeding habitats of harp seals across the North Atlantic [11]–[13]. The availability of ice-breeding habitats may affect reproductive success and survivorship of all age classes, although neonates are especially at risk to hypothermia, starvation, and crushing amidst moving ice [2], [6], [13]. Changes in the quantity and quality of ice cover will dictate where species are able to whelp (pup) [12] and may force seals to use sub-optimal areas potentially exposing individuals to other threats such as increased predation, disease and human interactions. Additionally, the timing of migration and pupping is strongly tied to ice extent across the entire range of the species [6]. Variability in ice cover remains an unquantified risk for harp seals, including increased juvenile mortality and changes in food availability [2], [13].

**Figure 1:**
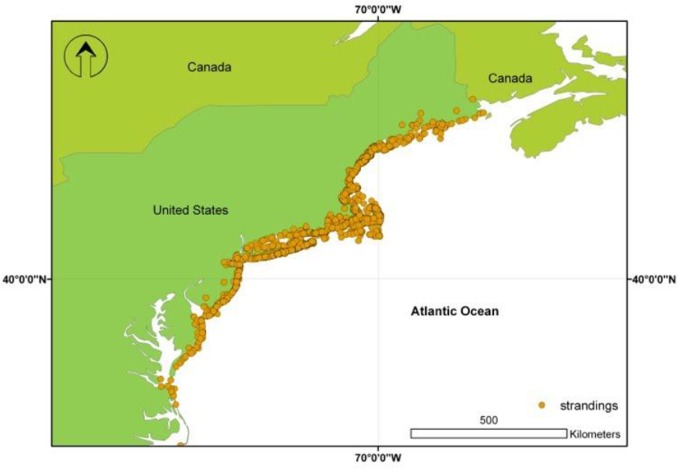
Harp seal strandings along the east coast of the United States: 1991–2010.

Recent years have seen an increase in harp seal strandings along the east coast of the U.S. [14]. Data collected from stranding networks show that since 1991 over 3,000 harp seals have stranded along the east coast from Maine to North Carolina (Figure 1). By March of 2011, five harp seals had been seen on the coasts of North Carolina and Virginia, more than ever previously recorded during this period [14]. It remains unclear what is causing this apparent increase in strandings. Previous studies have related neonate stranding rates to changes in sea ice cover in the breeding regions of harp seals [13], but a comprehensive analysis of harp seal strandings in the U.S. in relation to changing sea ice conditions and other biological factors has not been conducted.

In addition to environmental conditions, genetic fitness of the population may also influence harp seal stranding rates. In other pinniped populations, low genetic diversity in individuals has been associated with multiple measures of fitness, including survival [9], [15], disease susceptibility [16], [17], and reproductive success [18], [19]. The large harp seal population is thought to have relatively high genetic variation overall [9], but little is known about harp seal genetic diversity relative to historical times or across their full range, and high levels of exploitation (as many as 300,000 harp seals are harvested yearly in Canada) or by-catch can reduce genetic diversity [20]. At present, there are no studies assessing the potential effects of either climate-related or anthropogenic removals on the genetic variation found in seal populations, yet genetic diversity is an important factor influencing a population’s ability to adapt to a changing environment [21]. If anthropogenic removals or shifting climates have reduced genetic variation in harp seals, they may be at even greater risk to future changes in climate than predicted based on models of habitat change alone.

Genetic diversity can be assessed using microsatellite markers, which are presumably neutral genetic loci characterized by simple, tandem repeats that can be highly polymorphic. Microsatellites have previously been isolated in several phocid species, including harbor seals and grey seals, and these loci are known to have broad utility across other pinniped species [22], [23]. Of particular relevance to this study, Kretzmann et al. [9] used a set of these phocid microsatellite markers to examine genetic diversity of stranded juvenile harp seals from New York, demonstrating the use of microsatellites as a rough proxy for fitness in a species with high genetic variation and large population size. Kretzmann et al. [9] compared levels of genetic diversity between stranded seals that survived following rehabilitation efforts and those that did not, and found some evidence suggesting a relationship between higher genetic diversity and survival following rehabilitation. This association was not significant overall; however, their ability to detect an association of survival with genetic diversity was somewhat limited due to the confounding factor of time to rescue. In addition, the Kretzmann et al. [9] study addressed only fitness components involved in rehabilitation success and did not address the factors that cause strandings to occur. The latter would require a comparison of stranded seals to healthy representatives of the harp seal population. In this study of harp seal genetic diversity, by-caught harp seals are used as controls representing the healthy population [24].

In this study, we analyzed percent ice cover, demographics, and genetic diversity to determine their impact on stranding rates of harp seals along the eastern coast of the United States. We hypothesized: (1) that years of decreased ice cover would have higher numbers of strandings of neonates, while adult stranding rates would remain relatively constant, and (2) that stranded seals would have lower genetic diversity than by-caught seals.

## Methods

### Stranding Data Details

Stranding demographics were obtained from Level A data collected from stranding records along the eastern coast of the U.S. from 1991–2010 (Data available through NOAA Fisheries Office of Protected Resources – see http://www.nmfs.noaa.gov/pr/health/). We focused on two categories, age class and sex, which may contribute to variance in stranding rates. Age class was broken into two groups: yearling and adult. Any animal that did not have an age class was given one based on length. We created a range of lengths for yearlings from those with known age class, and any unknown seal that fell within that range ±1 standard deviation was classed as yearling and all others as adults. Within our yearling dataset, we also compared between stranding condition (alive vs. dead). All animals for which age class could not be determined or no sex was available were excluded from the analysis.

### Ice Cover Analysis

We assessed yearly percent ice cover for the month of February in the Gulf of St. Lawrence to provide an environmental context for harp seal stranding rates along the east coast of the U.S. from 1991 to 2010. Estimates of ice cover were derived from NASA Nimbus-7 Scanning Multi-channel Microwave Radiometer (SSMR) data (1979–1987) and Defense Meteorological Satellite Program (DMSP) Special Sensor Microwave/Imager (SSM/I) data for the Gulf of St. Lawrence as in Friedlaender et al. [11] and Johnston et al. [13]. Ice cover and stranding number anomalies were calculated in relation to the mean of the time series. Linear regressions were performed to assess the relationship of ice cover to the number of strandings for different sex and age classes of seals, and a least squares full factorial linear regression model was performed to assess the interaction of ice cover and significant demographic parameters. An analysis of covariance (ANCOVA) was performed to compare the regression slopes of our data (ice cover and total strandings) to the data from Johnston et al. (ice cover and dead yearling strandings) [13]. All statistical analyses of ice and stranding data were conducted in JMP 10.0.

### Sample Collection

Skin samples were collected from 106 harp seals that were either found stranded (N = 71) or by-caught (N = 35) along the east coast of the United States between 1992 and 2010. Samples were recovered mainly from Maryland, Maine, and Massachusetts by the Maryland Department of Natural Resources, the International Fund for Animal Welfare, and the NOAA Northeast Fisheries Science Center. Most samples were collected from alive or moderately decomposed animals. All skin samples were stored at −20°C in 95% ethanol until analyzed.

The stranded samples analyzed genetically are a subset of those used for the ice cover analysis described above. This subset is demographically representative (66.67% male, 33.33% female; 96.25% yearling, 3.75% adult) of the full dataset, but represents primarily light ice years (2000–2005), as expected due to the much higher number of available samples during those years.

### DNA Extraction and PCR

DNA was extracted from skin samples by digestion overnight in a 10% Chelex-100 (BioRad, Hercules CA) solution with 0.24 mg/ml of Proteinase K (Bioline, Taunton MA) at 60°C. Following digestion, the Proteinase K was denatured for 15 minutes at 100°C. We used 10 microsatellite primer pairs isolated from several species of pinnipeds and found to amplify polymorphic loci in harp seals (Table 1). Each forward primer was appended with a T3 tag (ATTAACCCTCACTAAAGGGA) to allow indirect labeling of PCR products with fluorescently labeled T3 primers.

**Table 1:** Microsatellite loci listed by species in which the locus was initially isolated.

Locus	Species	Reference
*Hl 8*	Leopard seal (*Hydruga lepotonyx)*	[41]
*Hl 15*		
*Pvc 9*	Harbor seals (*Phoca vitulina concolour)*	[22], [23]
*Pvc 16*		
*Pvc 19*		
*Hg 3.7*	Grey seals (*Halichoerus grypus*)	[23]
*Hg 4.2*		
*Hg 6.1*		
*Hg 8.10*		
*Hg 8.9*		

Polymerase chain reactions (PCR) were carried out in 20 µL reaction volumes. The reaction mix was as follows: 1.2 µL DNA, 1x PCR buffer (20 mM Tris pH 8.8, 50 mM KCl, 0.1% Triton X-100, 0.2 mg/mL BSA NEB purified), 2 mM MgCl_2_, 0.2 mM dNTPs, 0.1 µM forward primer (T3-tagged), 0.4 µM reverse primer, 0.4 µM T3 primer labeled with FAM, NED, PET, or VIC fluorescent dye, 0.2 µL Taq DNA Polymerase. The PCR profile included an initial denaturation at 94°C for 4 min, followed by 33 cycles of 94°C for 15 sec, 53°C for 15 sec, and 72°C for 30 sec, followed by a final extension at 72°C for 5 min.

### Sequencing and Genotyping

PCR products were diluted with 40 µL nanopure water and combined for multiplex genotyping. Genotyping reactions were carried out in a 13 µl reaction volume, including 4 µL of diluted PCR product (1 µL of each of 4 T3 fluorescent tag products), 0.04 µl of Orange DNA Size Standard (MC Labs) and 0.4 µg of sssDNA. The reactions were denatured at 94°C for 10 min before being run on an Applied Biosystems ABI 3730xl automated sequencer.

Genotyping was carried out using GeneMarker v1.8 (SoftGenetics). All runs for a marker were compiled together, and an allele panel was created in order to score each individual. The individual genotypes were visually checked to avoid any computer errors. Any non-existent, weak (reading below 100 relative fluorescent units, RFUs), and unreadable genotypes were removed from the analysis.

### Analysis of Molecular Data

Microsatellite loci were tested for deviations from Hardy-Weinberg equilibrium (HW), for linkage disequilibrium between pairs of loci, and for allele frequency differences among populations using GenePop (Web Version 4.2) [25]. For markers that were out of HW, MicroChecker was used to test for null alleles [26]. Outlier markers that could be linked to genes under selection were identified using Lositan Selection Workbench [27], [28]. Lositan evaluates the relationship between *F*_st_ and heterozygosity and identifies deviations from neutral expectations. The model was run with 10,000 simulations, assuming a stepwise mutation model and a forced ‘neutral’ mean *F*_st_.

*F*_st_, a measure of population differentiation that compares heterozygosity within subpopulations to that of the total sample [29], was calculated in Arlequin 3.11 [30] between stranded and by-caught seals to test if the two groups were sampled from a single population. STRUCTURE v2.2 was used to see if there was any evidence of population structure (i.e. Front and Gulf whelping patches) regardless of sampling location [31]. This program implements a Bayesian clustering approach to estimate population structure without requiring a priori designations of population membership. Independent runs for K = 1–3, where K is the number of populations, were performed using correlated allele frequencies and admixture models with 300,000 repetitions and a burnin of 50,000.

Two measures of genetic diversity, heterozygosity and mean *d* ^2^, were calculated and compared between the stranded and by-caught groups using t-tests. Mean *d* ^2^, calculated as the squared difference in allele size between two alleles at a locus averaged over many loci [32], can provide a better measure of recent inbreeding and population mixing than strict heterozygosity [15]. An individual with a low mean *d* ^2^ is more likely to be the progeny of related individuals and is more likely to experience inbreeding depression, and an individual with a high mean *d* ^2^ may be the progeny of more distantly related individuals [15]. We also calculated *d* ^2^ of individual loci for both stranded and by-caught seals. For the stranded group, we compared mean *d* ^2^ between males and females. Least squares full factorial linear regression models were performed in JMP 10.0 to investigate the relationship between ice cover, sample type (stranded/by-caught), sex, and mean *d* ^2^.

## Results

### Ice Cover and Total Stranding Rates

**Figure 2:**
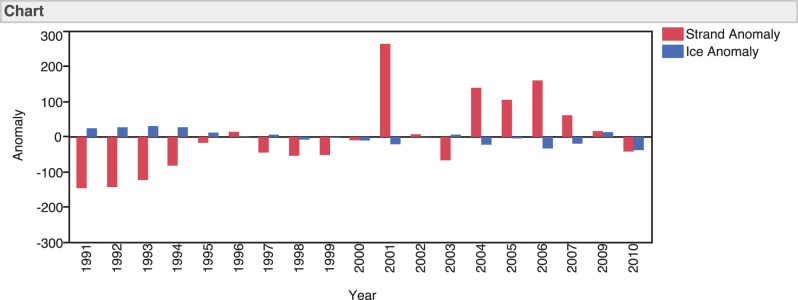
A time series comparing stranded seal anomaly and percent sea ice cover anomaly from 1991–2010.

During the study period, a total of 3,092 stranded harp seals were reported. The time series reveals a pattern in which years with light ice (generally lower than the overall mean) coincided with years of high numbers of stranded seals (Figure 2). From 1991 to 1995, ice cover was generally heavy and the number of stranded seals was relatively low. This was followed by a switch to lighter ice years and an increase in seal strandings after 1996. In 2001, eastern Canada experienced up to a 60% decrease in ice cover [2] coincident with the highest number of harp seal strandings in the database. Similarly, 2004 and 2006 were also light ice years with high numbers of stranded seals (Figure 2). The reverse was seen in 2003 when ice conditions were good and the number of strandings was relatively low. The pattern seems to be consistent with ice and strandings being out of sync until 2009. However, in the most recent years of the time series (2009 and 2010), the number of strandings was in sync with ice cover (Figure 2).

**Figure 3:**
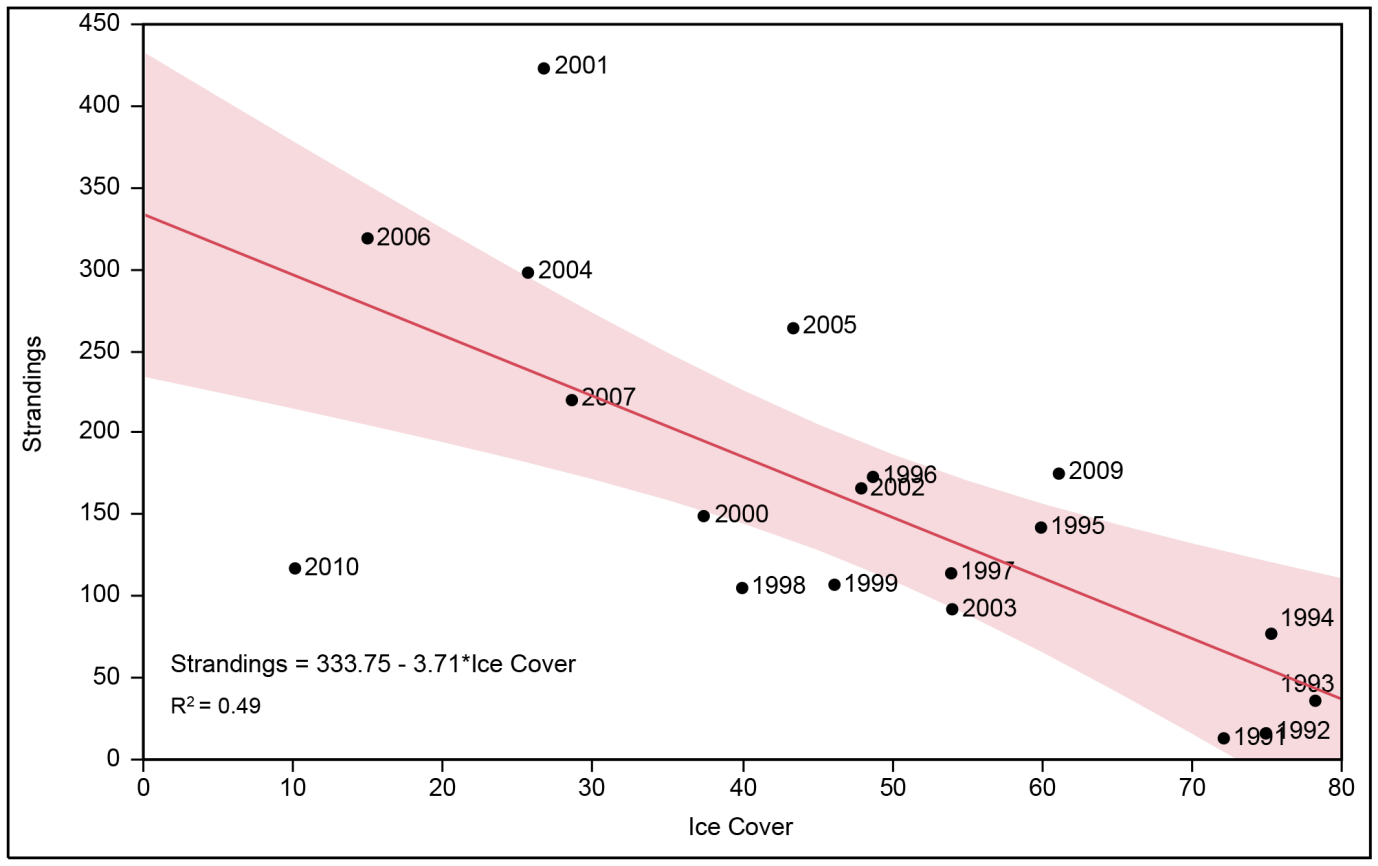
The relationship between percent sea ice cover and yearly harp seal strandings. The graph represents a linear regression between percent ice cover in February in the Gulf of St. Lawerence and yearly strandings along the Northeastern United States. The shaded area shows 95% confidence intervals.

**Figure 4:**
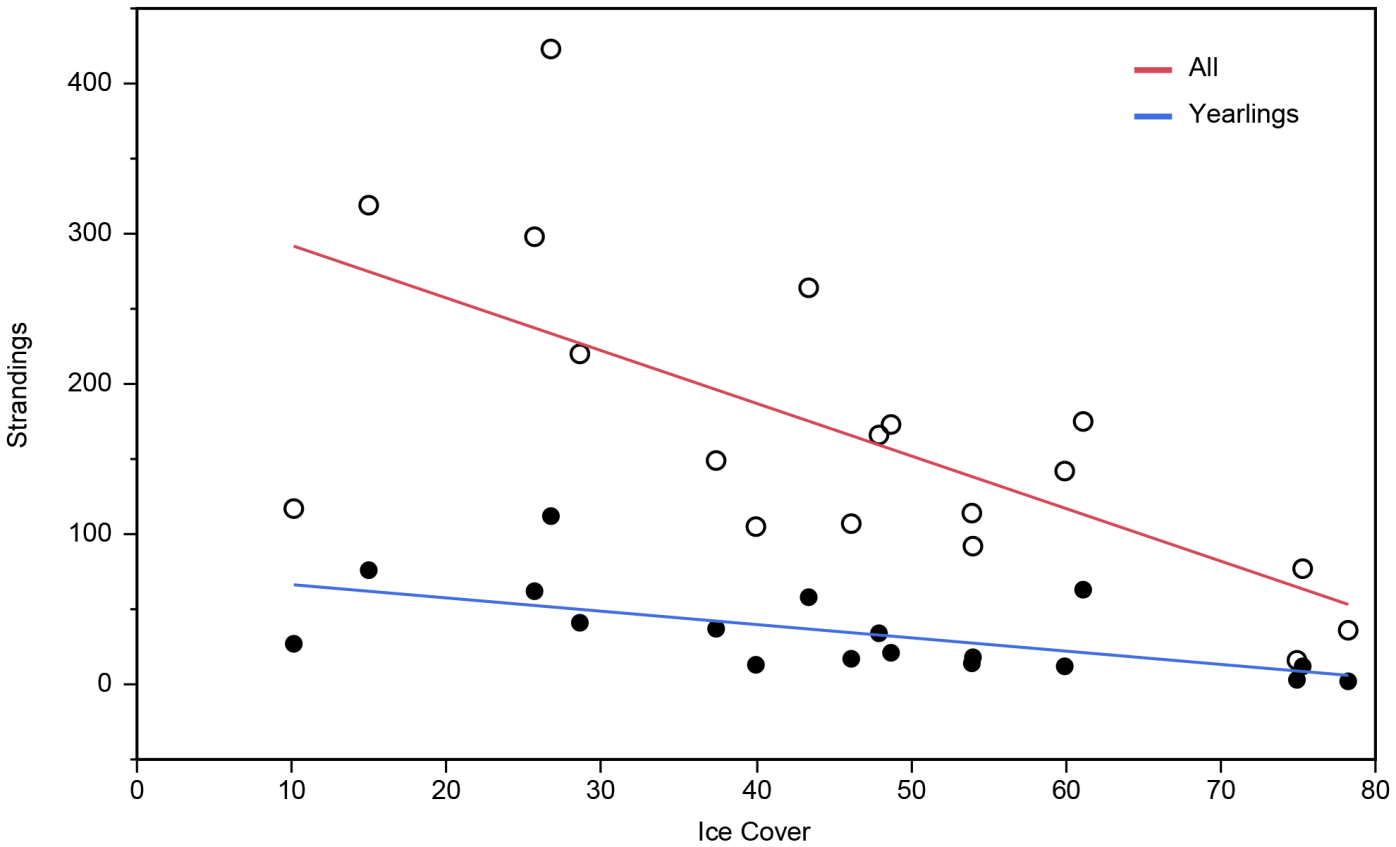
ANCOVA between linear regressions: percent sea ice cover vs. strandings (total and dead yearling strandings). The graph represents the ANCOVA between the linear regression of the present dataset (total strandings) and Johnston et al. [13](dead yearling strandings). Percent sea ice cover and total strandings (open circles and red line) and percent sea ice cover and dead yearling strandings (solid circles and blue line).

A linear regression of total harp seal strandings per year versus percent ice cover (Figure 3) showed a strong and significant negative correlation (Strandings = 333.75 - 3.71*Ice Cover, *R* ^2^ = 0.45, *P*<0.05). This is consistent with our hypothesis that years of decreased ice cover have greater numbers of strandings. Both 2009 and 2010 fell outside of the 95% confidence intervals with 2010 having the lowest percent ice cover (10%). The ANCOVA results revealed that the regression slope using the present dataset was significantly stronger (F = 23.24, *P*<0.0001) than that reported by Johnston et al. [13], indicating that stranding rates were higher in light ice years in the present analysis (Figure 4).

### Effects of Age Class, Stranding Condition, and Sex

**Figure 5:**
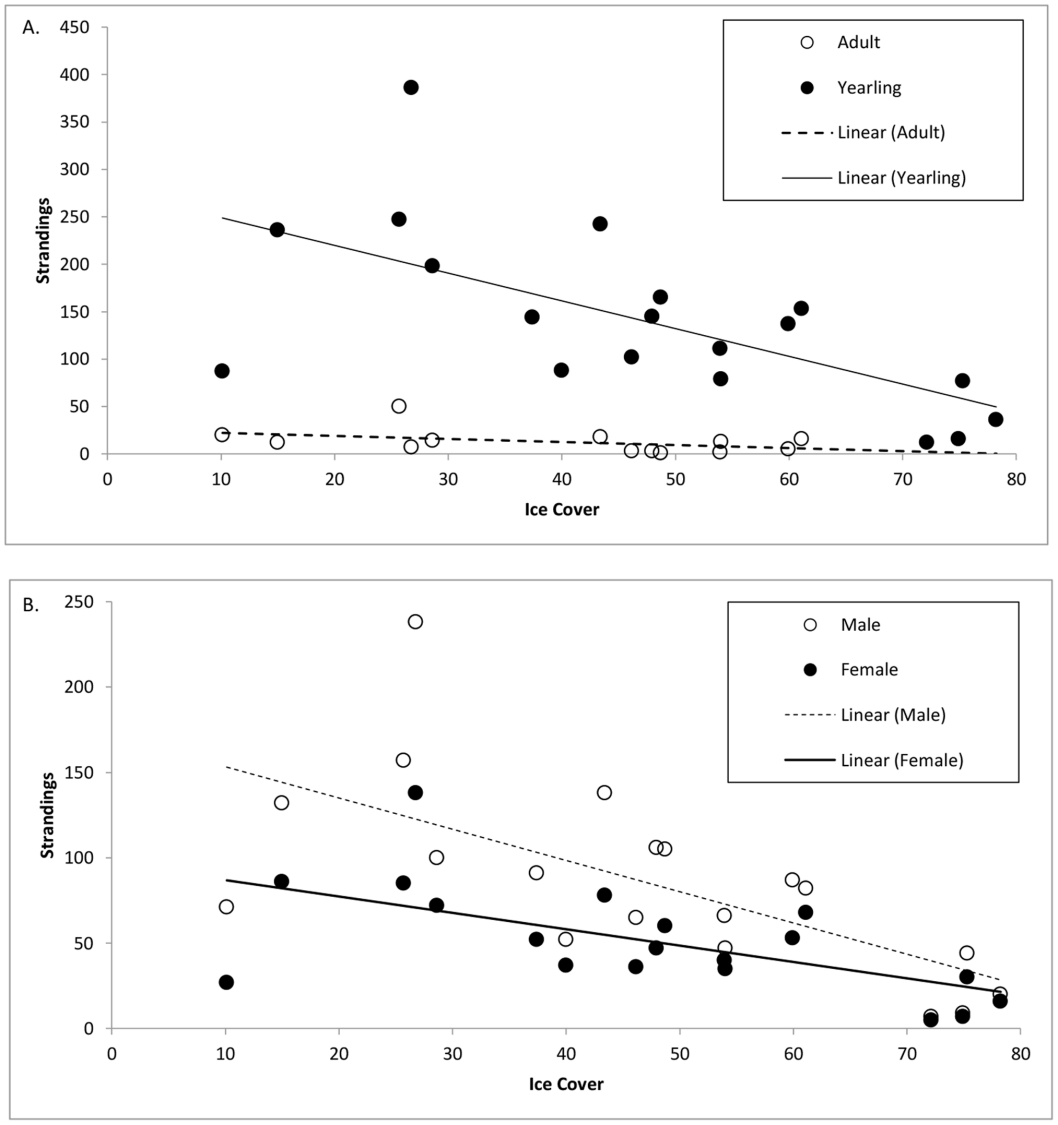
The relationship between percent sea ice cover and yearly strandings based on A. **age class and B. sex.** A. The graph represents a linear regression between percent ice cover in February and the number of yearly adult strandings (open circles and dashed line) and yearling strandings (solid dots and line). B. The graph represents a linear regression between percent ice cover in February and the number of yearly male strandings (open circles and dashed line) and female strandings (solid dots and line).

The total stranding rates were broken down into the different demographic categories, age class, yearling stranding condition, and sex, with per year and study period totals (Table 2). Across the entire time series, stranding rates of yearlings outnumbered that of adult seals. No significant relationship was found when regressing adult stranding rates against percent ice cover (*R^2^* = 0.18, *P = *0.15, Figure 5A). There was however a strong negative correlation and significant relationship between number of stranded yearlings and percent ice cover (*R^2^* = 0.41, *P*<0.05, Figure 5A), similar to that found by Johnston et al. [13] using a slightly shorter and more restrictive dataset. Within our yearling class, a majority of the seals stranded alive.

**Table 2:** Numbers of harp seal strandings along the eastern coast of the U.S. categorized by the demographic variables age class, yearling stranding condition, and sex.

	Age Class		Yearling Condition		Sex	
Year	Adult	Yearling	Alive	Dead	Male	Female
1991	–	12	12	–	7	5
1992	–	16	14	2	9	7
1993	–	36	32	4	20	16
1994	–	77	65	12	44	30
1995	5	137	111	26	87	53
1996	1	165	141	24	105	60
1997	2	111	93	18	66	40
1998	–	88	66	22	52	37
1999	3	102	77	25	65	36
2000	–	144	100	44	91	52
2001	7	386	266	120	238	138
2002	3	145	109	36	106	47
2003	13	79	64	15	47	35
2004	50	247	186	61	157	85
2005	18	242	181	61	138	78
2006	12	236	170	66	132	86
2007	14	198	153	45	100	72
2008	5	148	84	64	78	62
2009	16	153	89	64	82	68
2010	20	87	60	27	71	27
**Total**	169	2809	2073	736	1695	1034

For both males and females, there was a strong negative correlation and significant relationship between stranding rate and percent ice cover (males: *R^2^* = 0.44, *P*<0.05, females: *R^2^* = 0.37, *P*<0.05, Figure 5B). Overall, males stranded more frequently than females during the study period (t = −2.357, df = 30, 2-tailed *P*<0.05). In years of lighter ice cover, this difference between sexes was more drastic; there was a strong negative correlation between the ratio of male:female strandings and percent ice cover (*R^2^* = 0.33, *P*<0.01).

A least squares full factorial linear regression model confirmed the relationship between number of strandings, percent ice cover, and ratio of male:female strandings. The model significantly explained a large portion of the yearly variation in the number of harp seal strandings (*R^2^* = 0.55, F = 6.20, *P*<0.01). Ice cover had the largest effect on the number of strandings (F = 8.67, *P* = 0.01), and the interaction of ice cover and male:female ratio approached significance (F = 3.87, *P* = 0.07).

### Analysis of Molecular Data

A total of 106 harp seals (71 stranded and 35 by-caught) were genotyped at 7–10 microsatellite loci. There was no evidence of significant deviation from HW equilibrium (Table S1) or significant linkage disequilibrium between pairs of loci following sequential Bonferroni corrections for multiple tests [33]. Additionally, none of the 10 microsatellite loci showed evidence of non-neutrality based on the relationship between *F*_st_ and heterozygosity [29], [30]. These results indicate that the loci are appropriate for evaluation of neutral genetic diversity as a proxy for fitness.

There was no significant difference in overall allele frequency across all 10 loci between stranded and by-caught seals (χ^2^ = 29.507, df = 20, *P* = 0.08); however one locus (Hg 8.10) had significantly different allele frequencies between the two groups (*P*<0.01). More rare alleles were found in the group of stranded seals as expected due to the larger sample size. We found no significant differentiation between the stranded and by-caught groups (*F*_st = _0.00, *P = *0.68) and STRUCTURE v2.2 found no population structure in the complete dataset. This suggests that the individuals did not come from multiple genetically distinct breeding populations, which is consistent with previous studies that found no evidence for genetically distinct populations within the Northwestern Atlantic stock of harp seals [34], [35].

There was almost no significant difference in genetic diversity between stranded and by-caught seals. Heterozygosity at all 10 markers was high in most individuals, and the mean heterozygosity was not significantly different between the two groups (stranded: 0.843, by-caught: 0.821, t = −0.449, df = 18, 2-tailed *P = *0.66, Table S1). For six of the ten loci, the second measure of genetic diversity, *d* ^2^, was higher in by-caught seals than stranded seals (Table 3). However, the difference in *d* ^2^ between groups was only significant for one locus, Hg 8.9, and in this case *d* ^2^ was higher in stranded seals than by-caught seals. Overall, the mean *d* ^2^ for stranded and by-caught seals was not significantly different (stranded mean *d* ^2^ = 71.04, by-caught mean *d* ^2^ = 64.41, t = −0.754, df = 104, 2-tailed *P = *0.458).

**Table 3:** Mean *d^2^* by locus and P-values for t-test between stranded and by-caught seals.

	Mean *d* ^2^		
Loci	Stranded	By-caught	P-Value
Hg 3.7	49.82	63.73	0.398
Hg 8.9	101.02	21.38	**0.001**
Hg 8.10	62.73	87.52	0.457
Pvc 16	40.70	45.18	0.787
Pvc 9	35.88	53.14	0.255
Hl 8	53.97	41.07	0.342
Hg 6.1	64.42	56.87	0.643
Hg 4.2	78.23	82.88	0.820
Pvc 19	73.23	79.14	0.755
Hl15	149.45	116.24	0.414

Bolded values are significant.

Within the stranded samples, there was a significant difference in mean *d* ^2^ between males and females (male mean *d* ^2^ = 57.55, female mean *d* ^2^ = 92.56, t = 2.76, df = 24, 2-tailed *P*<0.05). The same comparison could not be conducted for by-caught animals because sex information was not available.

### Effects of Ice Cover, Sample Type, and Sex on Genetic Diversity

Percent ice cover (averaged for each year) had little to no effect on the genetic diversity of harp seals stranded or by-caught in a given year. The overall model of mean *d* ^2^ as explained by ice cover and sample type (stranded/by-caught) was non-significant and explained very little of the observed variation in mean *d* ^2^ (*R* ^2^ = 0.05, F = 1.76, *P* = 0.16); however, ice cover was observed to have a significant effect on mean *d* ^2^ (t = -2.13, *P*<0.05). Ice cover explained a very small but significant amount of the variation in mean *d* ^2^ among stranded seals (*R* ^2^ = 0.07, *P*<0.05), but did not explain any of the variation in by-caught seals (*R* ^2^ = 0.01, *P* = 0.59). This relationship was stronger for stranded male seals (*R* ^2^ = 0.10, *P* = 0.05) and absent in female stranded seals (*R* ^2^ = 0, *P* = 1.0), although subsetting the dataset by sex reduces the sample size and thus power of the analysis. Overall, the relationship between ice cover and genetic diversity observed in stranded seals was largely driven by a small number of individuals with high mean *d* ^2^ that stranded in years with lower ice cover.

## Discussion

The goal of our study was to assess the roles of ice cover, demographics, and genetic diversity on harp seal stranding rates along the eastern coast of the United States. We hypothesized that light ice years would correlate with high stranding rates of neonate harp seals, while adult stranding rates would remain more constant in the Northeastern United States. Secondly, we hypothesized that stranded seals would have lower genetic diversity compared to by-caught seals, representatives of a healthy group. Overall our data more strongly support our first hypothesis, suggesting that ice cover has a greater influence on harp seal stranding rates than genetic diversity. In addition, our analysis suggests that demographic factors, such as age and sex, also play a role in determining which animals from the population are more likely to strand.

There is considerable variation in the total number of harp seals stranding each year along the east coast of the U.S. from 1991–2010 (Figure 2). Some of this variation is clearly linked to sea ice cover where light ice years tend to have a greater number of harp seal strandings, confirming previous studies [13]; however, sex and age class also clearly play a role. For example, in both heavy and light ice years, the stranding record shows that yearlings are the most dominant age class to strand. Yearlings are particularly vulnerable to decreases in ice cover [2], [6], [13], which could largely explain the observed increases in seal strandings during light ice years. Decreased ice cover and early thawing will force pups into the water earlier, potentially before they are able to fully fend for themselves [12]. Yearlings initially rely on an ice-based food web which can be affected by both the duration and extent of ice cover [36]. Years with extremely light ice conditions have resulted in high neonatal mortality recently, resulting in extremely small (or perhaps non-existent) year classes [37], [38].

Overall, our stranded samples had a sex ratio of 1.6∶1 males to females, but this ratio differed depending on ice cover. A greater number of males relative to females stranded in light ice years; in 2010, the year with lowest percent ice cover in our study, we observed the highest ratio of male:female strandings (2.6∶1). From the subset of stranded animals we analyzed genetically, it is apparent that male seals which strand have on average lower mean *d* ^2^ than female seals that strand. This suggests that male seals may be more susceptible to the effects of low genetic diversity, but does not explain why females with higher genetic diversity strand. Male harp seals have a greater tendency to wander than female harp seals [6], which may affect both where the animals strand and the amount of physiological stress they experience related to travel. Regardless, the strong negative relationship with ice cover seen in both sexes illustrates that even though males may be the more prevalent sex seen stranded, changes in ice cover are impacting the entire population.

Other than the difference in mean *d* ^2^ observed between male and female stranded harp seals, our analyses show little evidence of an effect of genetic diversity on harp seal strandings. There was no significant difference in genetic diversity or allele frequencies between stranded and by-caught seals, which represented the healthy population, and there was little evidence of a relationship between ice cover and genetic diversity of stranded harp seals. These results are consistent with a previous study that found no difference in mean *d* ^2^ within stranded harp seals, comparing those that survived following rehabilitation to those that did not [9]. Several other studies have shown that the influence of genetic diversity on survival varies among causes of death [16], [17], which could not be considered here because cause of death was rarely determined or recorded. Even when considering this limitation, overall it appears that low genetic diversity is not a threat to most individuals in the Northwest Atlantic harp seal population.

The small number of seals (N = 15) that stranded with very high mean *d* ^2^ (>100) did almost all strand in years with lower percent ice cover (<45%). This observation might suggest that individuals with higher genetic diversity are stressed to the point of stranding during years of low ice cover, but not during years of average or high ice cover. However, we were limited in our ability to test this hypothesis by a lack of samples from heavy ice years. This limitation is largely a result of the lower number of stranded seals during heavy ice years. Future studies should target stranded seal samples from heavy ice years, when possible, in order to further test the relationship between sea ice cover and genetic diversity.

While this study focused on neutral genetic diversity, it is also possible that seals may strand due to genetic susceptibility in protein-coding genes. For example, variation within the major histocompatibility complex (MHC) has been associated with disease resistance in other pinniped species [9], [39] and potentially could play a more important role than neutral genetic diversity in this system. Interestingly, both studies of microsatellite diversity in stranded harp seals, the present study and that by Kretzmann et al. [9], found the same microsatellite locus, Hg 8.9, to have higher *d* ^2^ in the less “fit” group of harp seals (stranded seals and non-surviving seals, respectively). These findings may suggest that the microsatellite locus Hg 8.9 is linked to a gene inferring susceptibility in harp seals and warrants further study in addition to other candidate genes.

With the increasing rate of climate change it is necessary for researchers to understand the ability of species to adapt to changing conditions and to quantify their inherent resilience [5]. In order to survive, harp seals have to be able to adapt to changing ice cover in their breeding regions. Our results show high genetic diversity in both stranded and by-caught seals, suggesting that genetic diversity may not be a limiting factor in the adaptability of the species. However, it is difficult to predict whether or not seals will be able to adapt at a rate which keeps pace with rapid changes in ice cover.

As ice cover continues to fluctuate, we would expect the number of seal strandings to continue to vary negatively with ice cover, but that is not what is seen in the last two years of our dataset (Figure 2). In 2010, percent ice cover was the lowest in the dataset, but the number of strandings was close to the overall average. This decoupling of ice cover and stranding rates may represent an overall decline in the population, where there are fewer seals overall, and therefore fewer animals strand. It is more likely however that this change may reflect a shift in the phenology in this population of seals. Rosing-Asvid [40] provides initial evidence of a potential phenological shift in seals around Greenland, with seals staying in the area longer before heading off to whelping grounds. Further research and a longer time series of data are required to test this hypothesis.

In the meantime, managers need to begin to create conservation plans that incorporate shorter-term climate variability and longer-term climate change as major influences on marine species [36]. Recent studies have linked changes in ice cover in the breeding regions of harp seals across the North Atlantic to short-term climate variability [2], [11]–[13], but seasonal ice cover in these regions is decreasing at up to 6% per decade, regardless of the effects of the North Atlantic Oscillation [13].

Other species in the Arctic that rely on seasonal sea ice cover are also being affected [5], [36]. Hooded seals (*Cystophora cristata*) share some life history traits with harp seals and exhibit similar stranding patterns. Hooded seals have a smaller population along the east coast of Canada, but seem to be similarly affected by ice cover in their whelping areas. Hooded seals that breed in the West Ice region have declined by over 90% since the 1940s, coincident with a continual decline in sea ice in that region [13].

The available evidence suggests that Arctic ice cover will continue to decrease, and it will be crucial to know how those changes will affect not only the species that specifically rely on seasonal ice cover but also the system as a whole. Abundant high-latitude species, like harp seals, will be indicators of how the system is changing and what managers can do to conserve the species and resources of the Arctic system.

## Supporting Information

Table S1 **Summary of microsatellite loci used in this study, including observed heterozygosity (1^st^ column) and P-value for Hardy-Weinberg Equilibrium tests (2^nd^ column) within each group of harp seals.**
(DOCX)
