# Supplementary material for: Factors Affecting Harp Seal (Pagophilus groenlandicus) Strandings in the Northwest Atlantic
Source: PLoS One. 2013 Jul 17;8(7):e68779. doi: 10.1371/journal.pone.0068779 (PMC3714304; doi:10.1371/journal.pone.0068779)
Supplement: Table S1 — Summary of microsatellite loci used in this study, including observed heterozygosity (1st column) and P-value for Hardy-Weinberg Equilibrium tests (2nd column) within each group of harp seals. (DOCX) [file pone.0068779.s001.docx]

|  |  |  |  |  |  |  |  |  |  |  |  |  |  |  |  |  |  |  |  |  |  |
| --- | --- | --- | --- | --- | --- | --- | --- | --- | --- | --- | --- | --- | --- | --- | --- | --- | --- | --- | --- | --- | --- |
| **Locus** | Hg 3.7 |  | Hg 8.9 |  | Hg 8.10 | | Pvc 16 | | Pvc 9 |  | Hl 8 |  | Hg 6.1 |  | Hg 4.2 | | Pvc 19 | | Hl15 |  | Average H_O_ |
| **Stranded** | 0.794 | 0.02 | 0.825 | 0.20 | 0.955 | 0.82 | 0.667 | 0.01 | 0.794 | 0.42 | 0.821 | 0.12 | 0.864 | 0.06 | 0.901 | 0.62 | 0.969 | 0.58 | 0.894 | 0.70 | 0.848 |
| **By-caught** | 0.8 | 0.30 | 0.688 | 0.08 | 0.818 | 0.67 | 0.794 | 0.92 | 0.821 | 0.98 | 0.724 | 0.01 | 0.862 | 0.01 | 0.971 | 0.67 | 0.857 | 0.13 | 0.939 | 0.23 | 0.827 |
|  |  |  |  |  |  |  |  |  |  |  |  |  |  |  |  |  |  |  |  |  |  |

None of the deviations from Hardy-Weinberg remain significant following a table-wide sequential Bonferroni correction for multiple tests.
